# Assessment and Management of PTSD Among Children and Adolescents with ADHD as a Preventive Strategy for Suicidal Behaviours

**DOI:** 10.1192/j.eurpsy.2025.154

**Published:** 2025-08-26

**Authors:** H. Kerbage

**Affiliations:** 1 Developmental Psychiatry Team, Center for Epidemiology and Population Health (CESP) INSERM U1018, Paris-Saclay University, Paris; 2 Department of Child and Adolescent Psychiatry, Montpellier University Hospital, Montpellier, France

## Abstract

**Abstract:**

Children and adolescents with Attention-Deficit/Hyperactivity Disorder (ADHD) are at an increased risk of experiencing traumatic events and developing Post-Traumatic Stress Disorder (PTSD). The interplay between ADHD and PTSD presents unique diagnostic and therapeutic challenges, as symptoms such as emotional dysregulation, impulsivity, and attentional difficulties may overlap, complicating early identification and intervention. Notably, the presence of both ADHD and PTSD significantly heightens the risk of suicidal spectrum behaviors, underscoring the need for targeted clinical strategies. This presentation will explore current evidence on the association between ADHD, PTSD, and suicidal behaviors in youth, highlighting the neurobiological, cognitive, and psychosocial mechanisms that contribute to this heightened vulnerability. We will discuss practical approaches for screening PTSD in children and adolescents with ADHD, considering both clinical and psychometric tools tailored to this population. Additionally, we will examine intervention strategies that integrate trauma-focused care within ADHD management, including psychoeducation, cognitive-behavioral therapy (CBT), parent training, and pharmacological considerations.

**Disclosure of Interest:**

None Declared

